# Supplementary material for: The Utilization of an Aloe Vera Rind By-Product: Deep Eutectic Solvents as Eco-Friendly and Recyclable Extraction Media of Polyphenolic Compounds
Source: Antioxidants (Basel). 2024 Jan 26;13(2):162. doi: 10.3390/antiox13020162 (PMC10886410; doi:10.3390/antiox13020162)
Supplement: Supplementary file 1 [file antioxidants-13-00162-s001.zip › antioxidants-2840243-supplementary.pdf]

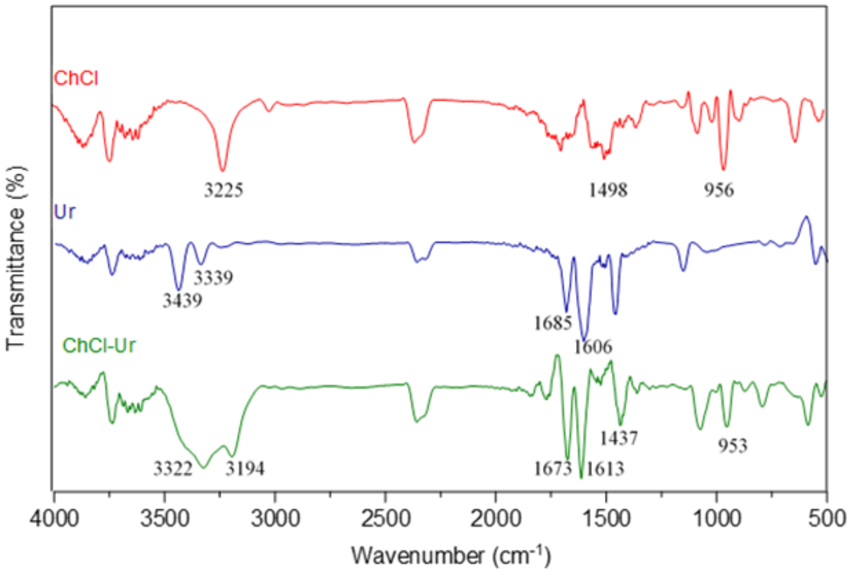

Figure S1. The FTIR spectra of ChCl, Ur and ChCl-Ur.

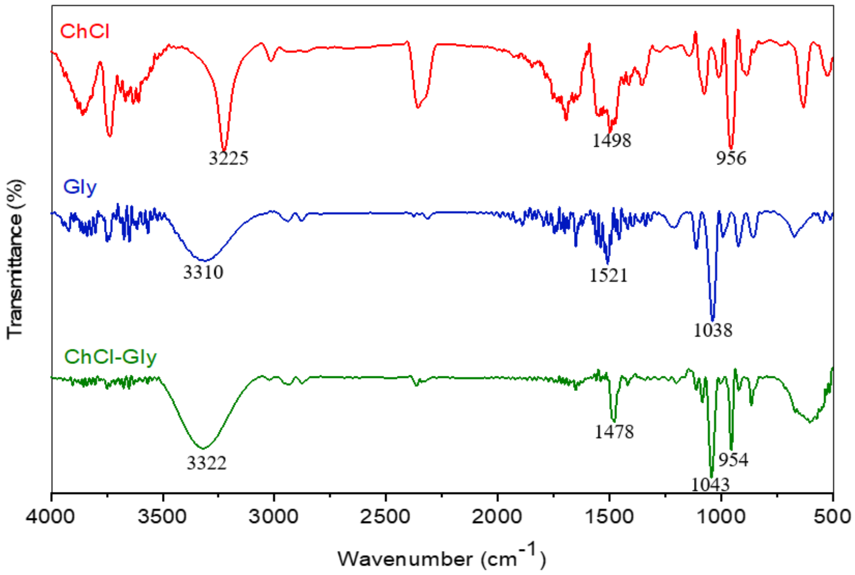

Figure S2. The FTIR spectra of ChCl, Gly and ChCl-Gly.

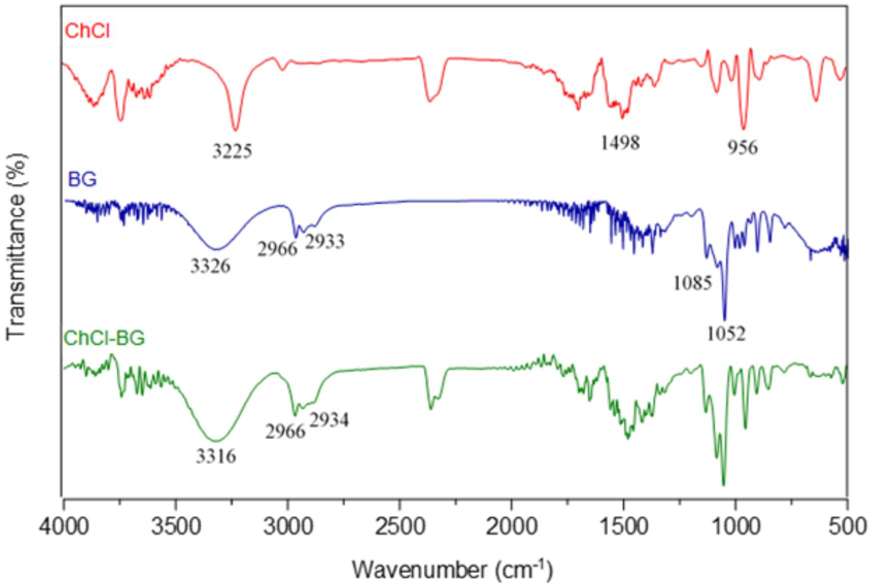

Figure S3. The FTIR spectra of ChCl, BG and ChCl-BG.

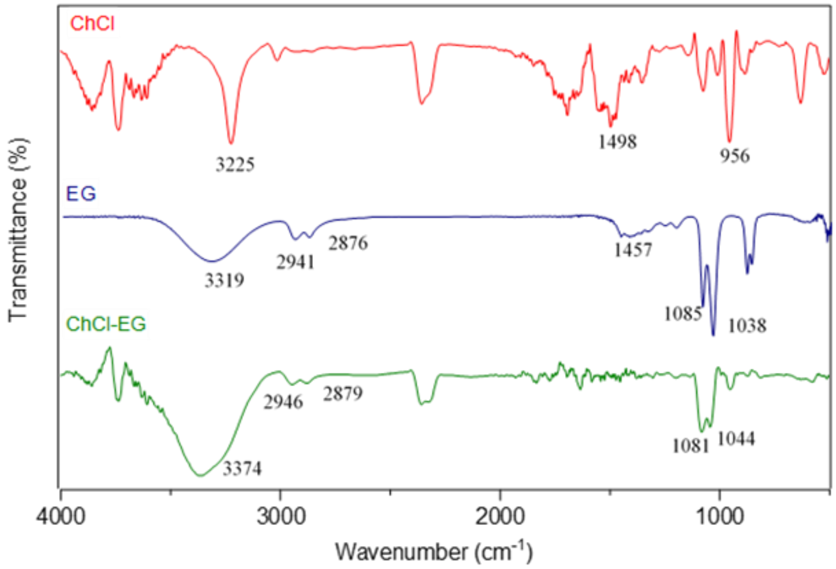

Figure S4. The FTIR spectra of ChCl, EG and ChCl-EG.

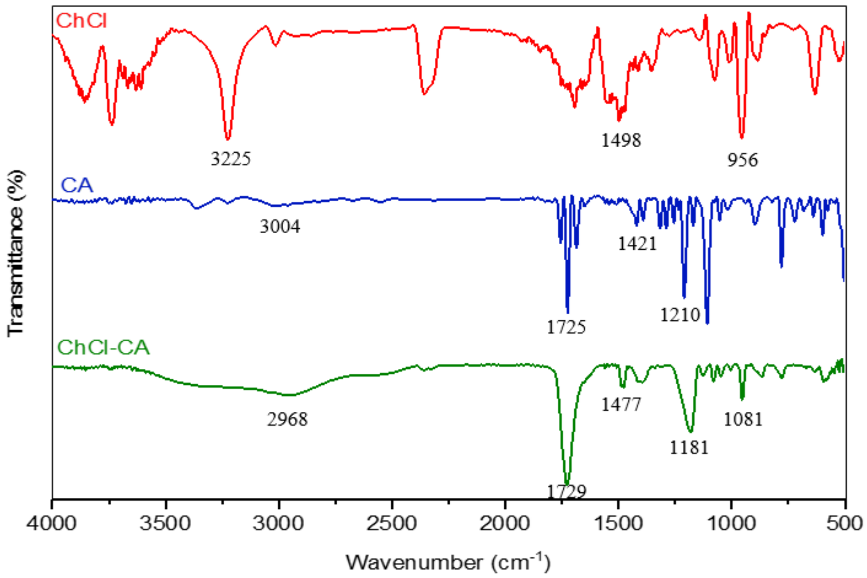

Figure S5. The FTIR spectra of ChCl, CA and ChCl-CA.

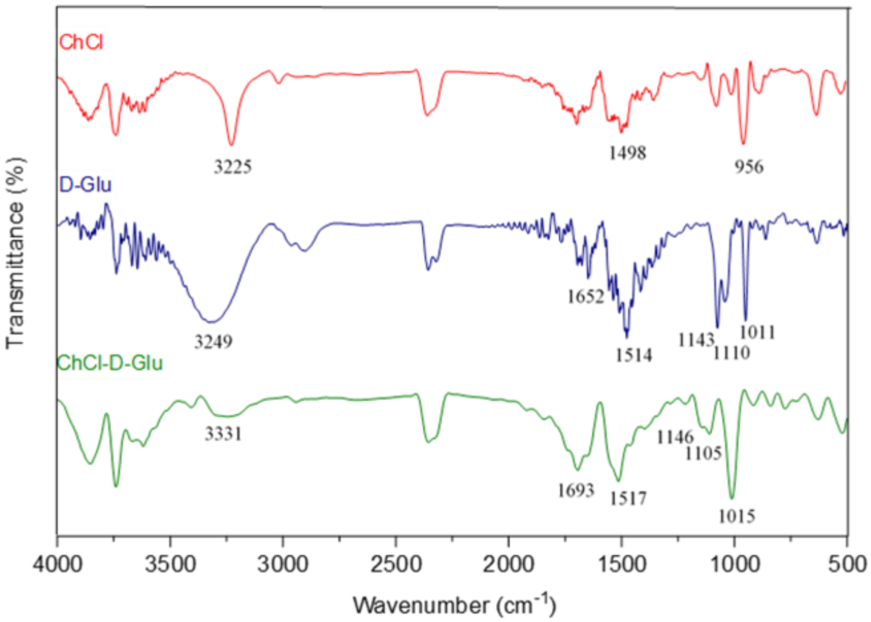

Figure S6. The FTIR spectra of ChCl, D-Glu and ChCl-D-Glu.

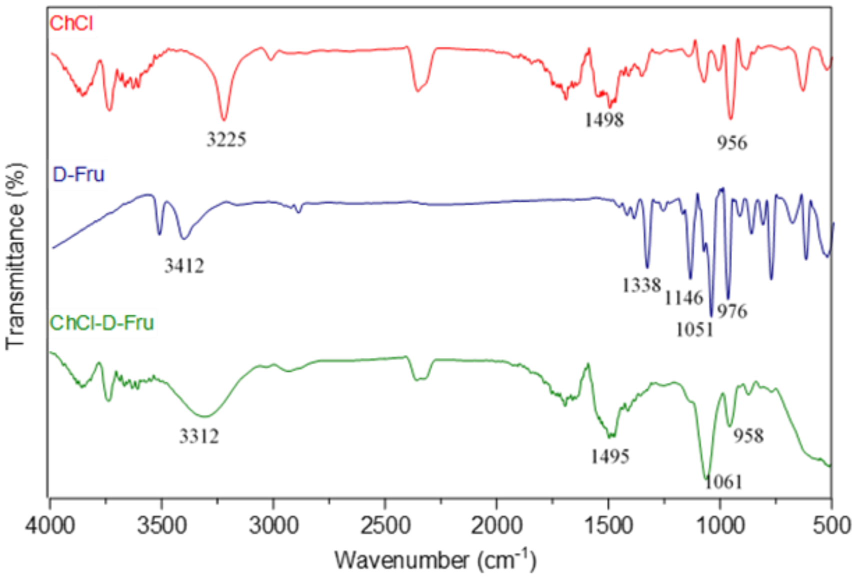

Figure S7. The FTIR spectra of ChCl, D-Fru and ChCl-D-Fru.

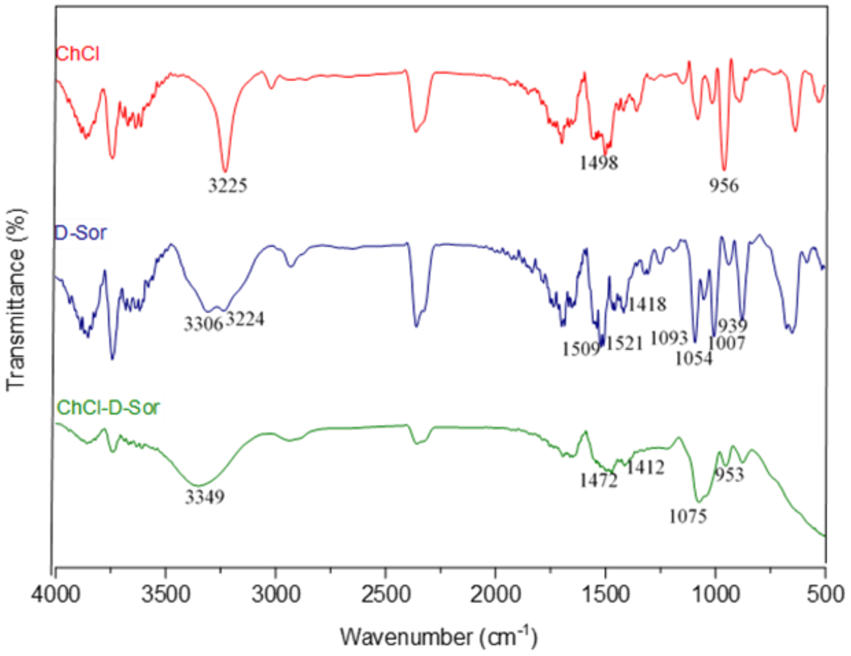

Figure S8. The FTIR spectra of ChCl, D-Sor and ChCl-D-Sor.

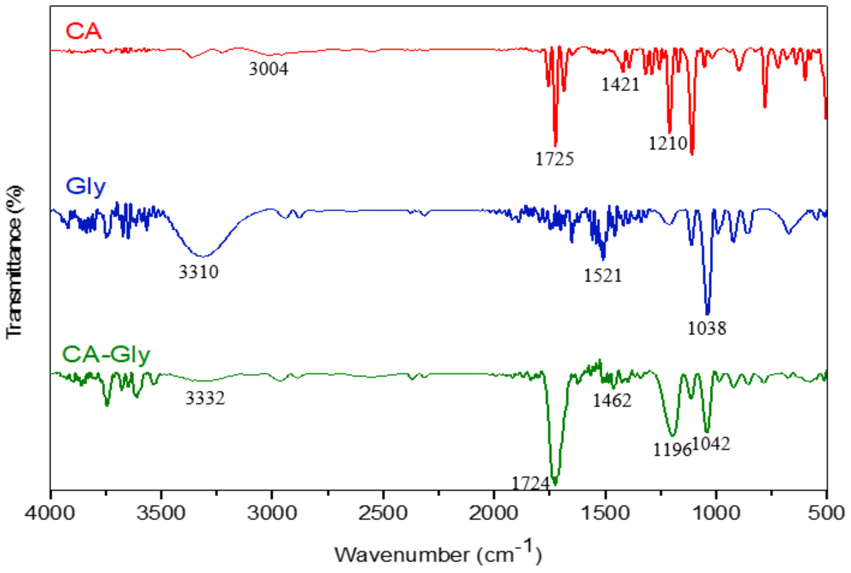

Figure S9. The FTIR spectra of CA, Gly and CA-Gly.

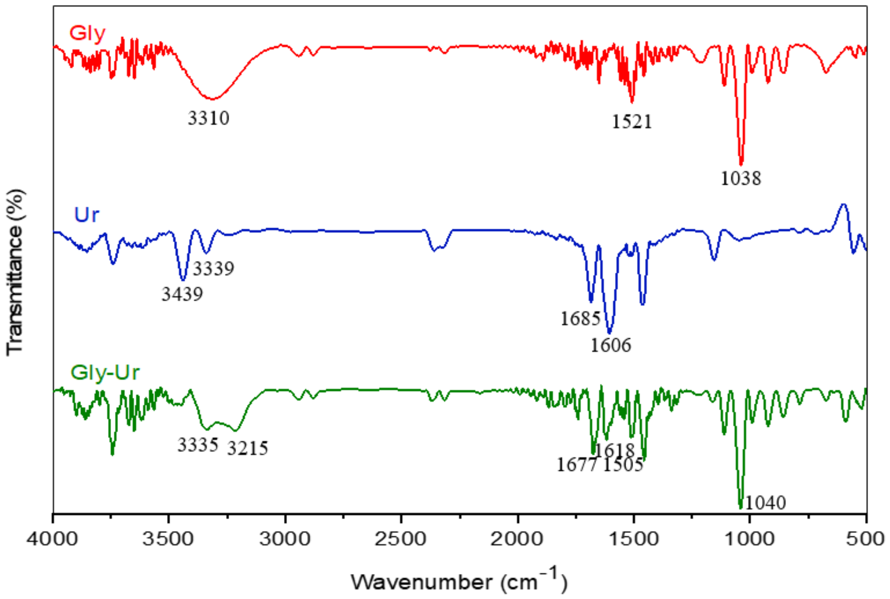

Figure S10. The FTIR spectra of Gly, Ur and Gly-Ur.

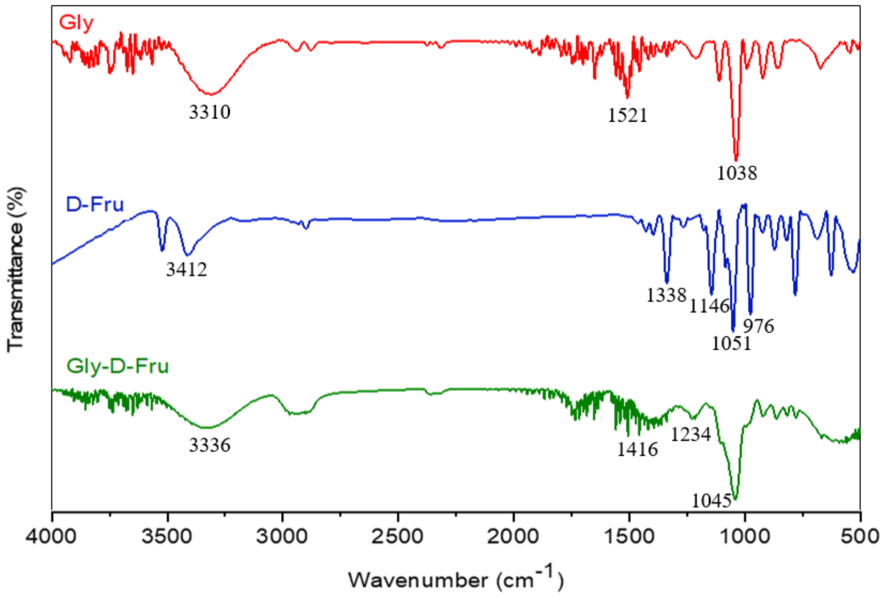

Figure S11. The FTIR spectra of Gly, D-Fru and Gly-D-Fru.

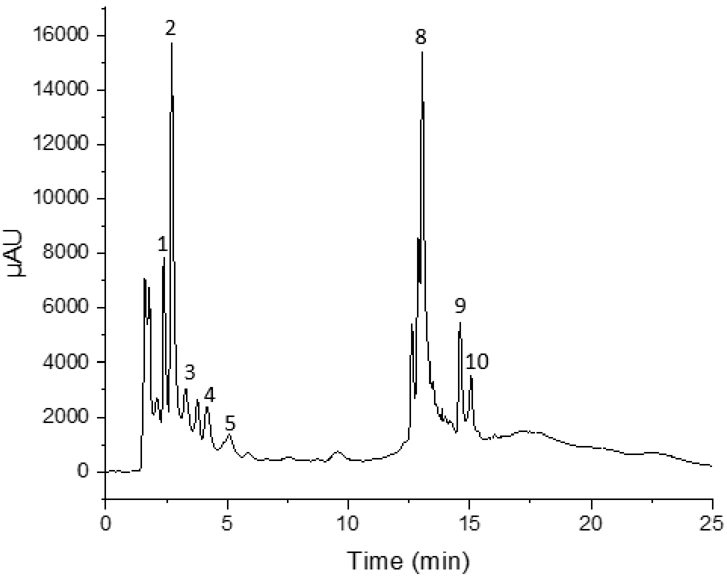

Figure S12. HPLC chromatograph of optimum aloe rind extract. 1) Gallic acid, 2) Catechin, 3) Epicatechin, 4) Caffeic acid, 5) Rutin, 8) Myricetin, 9) Quercetin and 10) Apigenin.

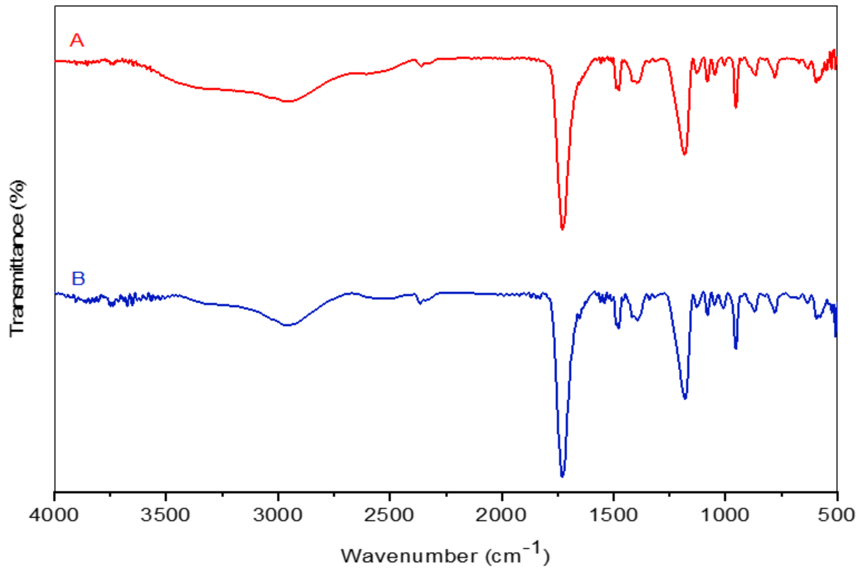

Figure S13. The FTIR spectra of A) ChCl-CA and B) obtained ChCl-CA by SPE.

Table S1. The three levels of independent variables of experimental design

Variables	Low level (-1)	Middle level (0)	High level (+1)
**Time**	10	15	20
**Solvent/solid**	100	150	200
**%DES**	60	70	80

Table S2. Validation of HPLC method.

Compounds	Linear range	Regretion equation	R^2	LOD (µg/ mL)	LOQ (µg/ mL)	Precision	
						Intraday	Interday
**Gallic Acid**	0.05-0.0001	y=99234156x-35103	0.999	0.7	2.1	1.00	0.92
**Catechin**	0.05-0.0001	y=13269933x-2012	0.999	0.3	0.9	0.95	2.53
**Epicatechin**	0.01-0.0001	y=16600346x+2404	0.998	0.3	0.8	1.04	1.47
**Caffeic Acid**	0.01-0.0001	y=55118151x+8713	0.997	0.3	1.0	0.47	1.96
**Rutin**	0.05-0.0001	y=23176073x-12698	0.998	0.9	2.8	0.55	1.80
**Sinapic Acid**	0.01-0.0001	y=39382578x+2882	0.997	0.4	1.1	0.33	1.92
**Quercitrin**	0.01-0.0001	y=12295377x-1169	0.999	0.1	0.3	0.42	1.84
**Myricetin**	0.01-0.0001	y=25427092x+2679	0.998	0.3	0.9	0.90	2.68

**Quercetin**	0.01-0.0001	y=34063703x+42715	0.998	0.3	0.9	0.32	1.67
**Apigenin**	0.01-0.0005	y=19529158x-1178	0.999	0.2	0.7	0.62	1.34
